# Inflammatory arthritis in systemic sclerosis is associated with elevated C-reactive protein and requires musculoskeletal ultrasound for reliable detection

**DOI:** 10.3389/fmed.2022.933809

**Published:** 2023-01-18

**Authors:** Daria Feldmann, Ilona Jandova, Ursula Heilmeier, Florian Kollert, Reinhard E. Voll, Stephanie Finzel

**Affiliations:** ^1^Department of Rheumatology and Clinical Immunology, Medical Center – University of Freiburg, Faculty of Medicine, University of Freiburg, Freiburg, Germany; ^2^Department of Anesthesiology, Krankenhaus Porz am Rhein, Cologne, Germany; ^3^Department of Rheumatology and Immunology, University Hospital of Bern, University of Bern, Bern, Switzerland

**Keywords:** systemic sclerosis, arthritis, CRP, musculoskeletal ultrasound, imaging

## Abstract

**Objectives:**

About 25% of patients with systemic sclerosis (SSc) have elevated C-reactive protein (CRP) levels. Specific causes of CRP elevation are unknown so far. We aimed to investigate whether inflammatory arthritis is associated with CRP elevation. Furthermore, we evaluated the sensitivity and specificity of clinical examination compared to musculoskeletal ultrasound (MSUS) for detection of arthritis.

**Methods:**

Sixty-five patients with SSc (51 females) were enrolled and allocated into a CRP-positive (CRP+, *n* = 20; CRP elevated for at least two years prior to enrollment) and a CRP-negative (CRP−; *n* = 45) cohort. All patients were examined clinically (modified Rodnan Skin Score, mRSS; swollen/tender joint count 66/68), received a comprehensive MSUS of their hands and feet, as well as laboratory testing (antibody status; CRP). Statistical analyses were performed using non-parametrical tests without adjustments.

**Results:**

Patient with a disease duration <3 years had higher CRP levels (*p* = 0.042). Anti-centromere antibodies dominated in CRP- patients (p = 0.013), and anti-Scl70 antibodies in CRP + patients (*p* = 0.041). Joint effusion and B-mode synovitis prevailed in male (*p* < 0.00001; *p* < 0.0001) and CRP + (*p* = 0.001; *p* < 0.00001) patients. Power Doppler (PD)-synovitis predominated in patients with diffuse SSc (*p* = 0.0052). Joint effusion and B-/PD-synovitis were mostly confined to wrists, MTPs and talo-navicular joints. Compared to MSUS, sensitivity of clinical examination was as low as 14.6%; specificity was 87.7%. Sensitivity was reduced by the presence of soft tissue edema or a mRSS > 10.

**Conclusion:**

Arthritis is more frequent in CRP + compared to CRP- SSc patients. Compared to MSUS sensitivity of clinical examination is low for the detection of arthritis; this is likely due to skin fibrosis and soft tissue edema. Therefore, regular monitoring via MSUS should be considered as routine assessment in SSc patients.

## Introduction

Systemic sclerosis (SSc) is a chronic inflammatory connective tissue disease characterized by an increased activation of the immune system, vasculopathy, as well as exuberant fibrosing processes affecting internal organs and skin (1). In about 25% of patients with SSc, elevated C-reactive protein (CRP) levels can be observed (2). Patients with constantly elevated CRP levels over time may have a different disease phenotype than patients with normal CRP levels. Mitev et al. (3) described an association between elevated CRP levels and a more severe disease progress. The specific cause of the CRP increase and the role of CRP in the pathogenesis of SSc are, however, unknown so far.

We hypothesized, that arthritis might contribute to CRP elevation. Therefore, we investigated SSc patients with and without elevated CRP levels for prevalence and localization of arthritis. Joint count 66/68 is an accepted method to clinically quantify arthritis. Musculoskeletal ultrasound (MSUS) and magnetic resonance imaging (MRI), however, are known to have a greater sensitivity for detecting subclinical arthritis than clinical examination (4–8). MSUS is a sensitive method to even detect subclinical inflammatory changes, which might on one hand imply the risk of overrating non-significant findings. It therefore requires some training in order to be able to avoid potential pitfalls such as false positive ratings. On the other hand, MSUS is a well-evaluated imaging method that has the advantage over MRI of providing highly sensitive information in B- and PD-mode of the patient in a short time and without additional burden or the use of contrast agents. Moreover, MSUS is much less costly compared to MRI. We thus evaluated the sensitivity and specificity of clinical joint assessment compared to MSUS.

## Patients and methods

### Patients

Sixty-five consecutive patients fulfilling the ACR/EULAR 2013 classification criteria (9) were recruited from the outpatient clinic at the Department of Rheumatology and Clinical Immunology, University Medical Center, Freiburg, Germany, and included in our cross-sectional study. CRP and antinuclear antibodies (ANA on Hep2 cells) and ENA including anti-Scl-70, anti-CENP A, anti-CENP B, anti-Sp100, anti-PML, anti-PM-Scl100, anti-PM-Scl75, anti-RP11, anti-RP155, anti-gp210, anti-PCNA, anti-SS-A, anti-Ro52, anti-SS-B, anti-nRNP/Sm, anti-Sm, anti-Mi2α and β, anti-Ku, anti-nucleoseome, anti-histone, anti-dsDNA anti-DFS70 antibodies using a line blot (Euroimmune, Lübeck, Germany) were analyzed as part of the routine assessment. Joint swelling and pain were recorded using the joint count 66/68, performed by an experienced rheumatologist (IJ) who was blinded to the CRP status of the study participants. Musculoskeletal function was assessed using the Health Assessment Questionnaire (HAQ). Arthralgia defined as non-mechanical pain in the joints as well as patient global health were assessed on a visual analog scale (VAS) ranging from 0 to 100 mm. Skin involvement was measured by the same experienced rheumatologist (IJ) using the modified Rodnan Skin Score (mRSS) (10). Furthermore, all patients received MSUS assessment as described below. Information on organ involvement and immunomodulatory medication was retrieved from the patient charts. Following clinical examination and assessment, as well as blood sampling, all patients received MSUS the same day by an experienced rheumatologist and ultrasonographer (SF) who was blinded to the patient’s CRP status and clinical examination results.

The study was approved by the Freiburg Institutional Review Board (386/17). The study was conducted according to ICH/GCP (in compliance with the declaration of Helsinki). All patients gave written informed consent prior to any study related measures.

### Group Assignment, inclusion and exclusion criteria

Patients were assigned to the CRP positive (CRP+) or CRP negative (CRP−) cohorts according to their CRP status over the last two years preceding study enrollment. The cut-off value for the highly sensitive CRP used in this study was <5 mg/L. CRP-status was deemed positive or negative if at least 75% of the CRP values were positive (>5 mg/L) or negative (<5 mg/L) in at least three half-yearly visits within the last two years. Confounding conditions such as the presence of an infection, trauma or intervention (antibiotics, etc.) were accounted for insofar as patients with other reasons for CRP elevation than SSc were not eligible for study participation. In order to minimize any potential therapy bias, patients were only included into the study if they had been on a stable therapy for at least one year prior to the study visit. Patients positive for anti-citrullinated protein antibodies (ACPA), with rheumatoid factor >25 IU/ml, diagnosed with an overlap syndrome or with myositis were excluded from study participation.

### Ultrasonographic evaluation

For all ultrasound scans an Esaote MyLab Twice ultrasound machine was used (Esoate, Genoa, Italy), and all scans were performed by the same physician (SF) with 9 years of experience in MSK ultrasound. All musculoskeletal scans were obtained using an 18 MHz linear array at 10-18 MHz in B-mode and at 10.2 MHz in Power Doppler (PD) mode (750 PRF).

In detail, bilateral wrists, metacarpophalangeal joints (M), proximal interphalangeal joints (PIPs), distal interphalangeal joints (DIPs), talonavicular and upper ankle joints as well as metatarsophalangeal joints (MTPs) were examined and evaluated in all patients to get an extensive image of the patient’s joint involvement. The wrists, MCPs, PIPs, and DIPs were scanned both at dorsal and palmar sites.

Each joint was examined for presence of effusion, synovial hypertrophy in B-mode and synovial hypervascularization in PD-mode in two perpendicular planes. Classification characteristics have been adapted according to the definitions outlined in earlier publications (11, 12): Joint effusion was defined as abnormal hypoechoic or echo-free intraarticular tissue that is displaceable and compressible but has no Doppler signal. Synovial hypertrophy was defined as an abnormal hypoechoic intraarticular tissue that is not displaceable and poorly compressible. Effusion and synovial hypertrophy were quantified in B-mode. The PD-mode provides a representation of the microvascular blood flow that is elevated in synovitis, thus allowing the distinction between active inflammation and hypertrophy alone. Joint effusion, as well as synovitis in B-mode and PD-mode were quantified using a 4-graded semi-quantitative scale for each item (0-3: 0 = absent, 1 = mild, 2 = moderate, 3 = severe) (13, 14). Exemplary ultrasound images and their grading are displayed in Supplementary Figures 1A–P.

## Statistical evaluation

For statistical analysis, the Mann–Whitney U test was used to compare age, disease duration, severity and frequencies of ultrasound findings and to compare clinical scores in the two cohorts (CRP+ and CRP−). The Spearman rank correlation test was carried out to compare the results of the clinical and ultrasound examination. R-values > 0.1–0.5 indicate a moderate and >0.5–1 a strong correlation. The remaining calculations were carried out employing the Fisher’s exact test, whereby binary distributed characteristics can be examined in two different cohorts. The data was either presented as mean value ± standard deviation, or relative frequencies (%) with information on the total number. The level of significance was set at *p* < 0.05. Statistical analyses were guided/supervised by expert biostatisticians (MK, Ortmann Statistik©; see section “Acknowledgments”).

## Results

### Patient’s characteristics

**FIGURE 1:**
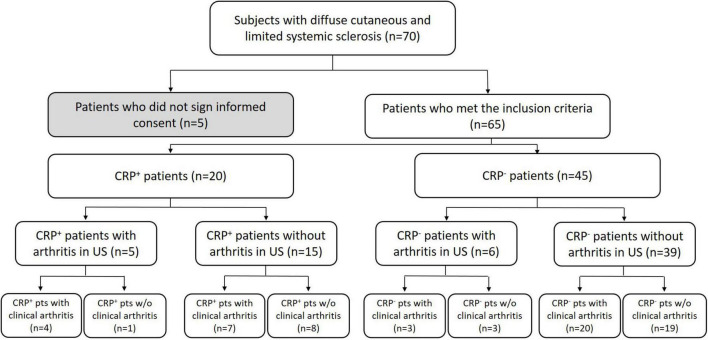
STROBE-diagram of group allocation as well as inclusion and exclusion criteria are shown.

Sixty-five patients were enrolled into this cross-sectional study; see Figure 1 for details of cohort allocation and inclusion/exclusion criteria. The CRP+ and CRP− cohorts did not significantly differ in terms of duration of illness, mean age, age of onset, gender or group size of subtypes, i.e., limited vs. diffuse SSc. However, when considering only patients with a disease duration of <3 years, this subgroup had significantly higher CRP levels (*p* = 0.042) compared to those with longer disease durations. A similar observation has been reported previously by Muangchang (2). For details of patients’ characteristics please see Table 1.

**TABLE 1:** Baseline characteristics of all study participants at the study visit.

	Total	CRP + cohort	CRP – cohort	*P*-value
	(*n* = 65)	(*n* = 20)	(*n* = 45)	
**Age at study visit (in years ± SD)**	58.38 ± 13.54	59.25 ± 12.98	58.00 ± 13.90	0.984
**Median age (range)**	59 (r: 23–82)	63 (r: 36–82)	57 (r: 23–80)	
**Gender (f/m)**	51/14	13/7	38/7	0.105
**Age of SSc diagnose (in years ± SD)**	48.09 ± 13.79	50.35 ± 12.26	47.09 ± 14.43	0.453
**Median (range)**	47 (r: 17–81)	50,5 (r: 30–81)	46 (r: 17–77)	
**Type of SSc (limited/diffuse)**	37/28	11-Sep	28/17	0.278
**Disease duration (in months ± SD)**	123.62 ± 98.94	107.2 ± 114.6	130.9 ± 91.6	0.093
**median (range)**	104 (r: 0–393)	60 (r: 2–386)	108 (r: 0–393)	
**Frequency of organ involvement *n* (%)**	42 (64.6%)	15 (75.0%)	27 (60.0%)	0.276
**Frequency of esophagus inv. *n* (%)**	29 (44.6%)	10 (50.0%)	19 (42.2%)	0.598
**Frequency of lung involvement *n* (%)**	26 (40.0%)	10 (50.0%)	16 (35.6%)	0.289
**Frequency of gastrointestinal involvement *n* (%)**	4 (6.1%)	1 (5%)	3 (6.7%)	1
**Frequency of heart involvement *n* (%)**	2 (3.1%)	1 (5%)	1 (2.2%)	0.524
**Frequency of kidney involvement *n* (%)**	1 (1.5%)	0 (0%)	1 (2.2%)	1
**Usage of immunosuppressants *n* (%)**	25 (38,5%)	9 (45%)	16 (35,6%)	0.583
**Glucocorticoids *n* (%)**	14 (21.5%)	8 (40%)	6 (13.3%)	**0.023**
**Hydroxychloroquine *n* (%)**	12 (18.5%)	0 (0%)	12 (26.7%)	**0.012**
**Mycophenolate mofetil *n* (%)**	11 (16.9%)	3 (15%)	8 (17.8%)	1
**Methotrexate *n* (%)**	4 (6.1%)	2 (10%)	2 (4.4%)	0.581
**Azathioprine *n* (%)**	2 (3.1%)	1 (5%)	1 (2.2%)	0.524
**Rituximab *n* (%)**	2 (3.1%)	1 (5%)	1 (2.2%)	0.524
**Cyclophosphamide *n* (%)**	1 (1.5%)	1 (5%)	0 (0%)	0.308

CRP− status was deemed positive or negative, if at least 75% of the CRP values were positive (>5 mg/L) or negative (≤5 mg/L) in at least three half-yearly visits within the last 2 years. The cut-off value for the highly sensitive CRP used in this study was <5 mg/L. CRP, C-reactive protein; CRP+, CRP-positive cohort; CRP−, CRP-negative cohort; SD, standard deviation; f female; m, male; n, number; SSc, systemic sclerosis. Organ manifestations: lung, heart, kidney, liver, gastrointestinal involvement. Immunosuppressive treatments: methotrexate, azathioprine, leflunomide, mycophenolate, rituximab, cyclophosphamide, glucocorticoid.

### Autoantibodies

Thirty-six patients had anti-centromere antibodies (55.4%), and 18 anti-Scl-70 antibodies (27.7%), two patients had anti-centromere and anti-Scl-70 antibodies (3.1%); in nine patients neither anti-centromere nor anti-Scl-70 antibodies (13.8%) were present. When comparing patients positive for anti-Scl-70 antibodies to patients positive for anti-nucleosome antibodies, we found that anti-nucleosome antibodies were more frequent in CRP- patients (*p* = 0.013), whereas in CRP+ patients anti-Scl-70 antibody-positivity was more common (*p* = 0.041).

### Health assessment questionnaire and joint pain

SSc-patients with tender joints in clinical examination were significantly compromised in all HAQ-domains of joint function and mobility, despite the domains of dressing/grooming and activity. Patients with tender joints also reported significantly higher values on the VAS for joint pain and their disease intensity, see Table 2.

**TABLE 2:** Correlations between tender joints on clinical examination versus total HAQ score, singular domains of HAQ, VAS pain and VAS PGH are shown.

	Spearman’s Rho (*r*)	*P*-value
**Dressing/grooming**	0.18288	0.145
**Arising**	0.34598	**0**.**005**
**Eating/drinking**	0.38362	**0**.**00161**
**Walking**	0.26885	**0**.**030**
**Hygiene**	0.2911	**0**.**019**
**Reach**	0.4356	**<0**.**001**
**Grip**	0.39242	**0**.**001**
**Activities**	0.21161	0.091
**Total HAQ score**	0.3487	**0**.**004**
**VAS pain**	0.53018	**0**.**00001**
**VAS patient global health**	0.3884	**0**.**001**

HAQ, health assessment questionnaire; VAS, visual analogue scale. Bold *p*-values are significant. *R*-values >0.1–0.5 indicate a moderate and >0.5–1 a strong correlation.

### Joint evaluation

Localization and prevalence of joint effusion and arthritis in CRP+ and CRP− patients.

In general, joint effusion as well as synovitis in B- and PD-mode was most frequently found in the first and second MTP joints, the talo-navicular joints as well as the wrists.

In detail, joint effusion was significantly more frequent in CRP + patients in the talo-navicular joints (*p* = 0.0181), and B-mode synovitis was more frequent in the MTP I (*p* = 0.021) and MTP III joints (*p* = 0.0075) compared to the CRP- patients.

**FIGURE 2:**
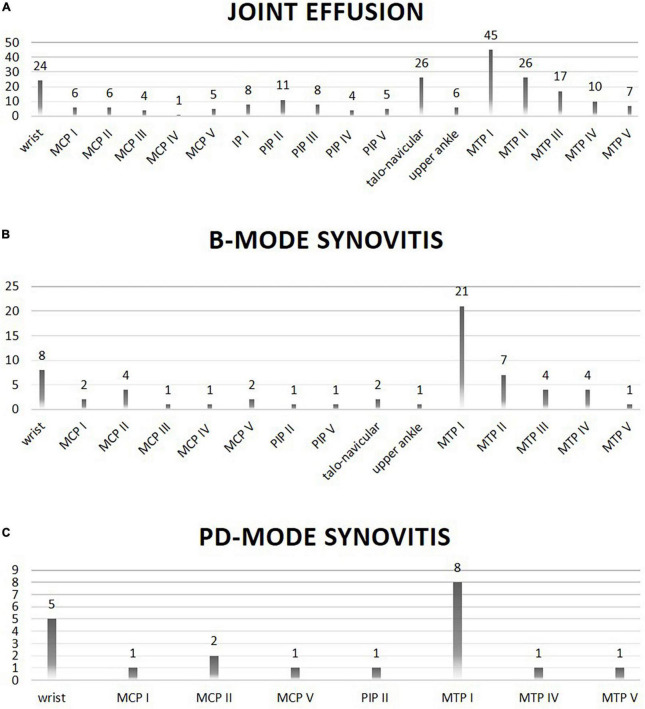
Location (X-axis) and frequency (Y-axis; absolute numbers) of joint involvement on ultrasound evaluation is shown. Panel **(A)** shows joints with effusion on ultrasound evaluation. Panel **(B)** shows joints with B-mode synovitis on ultrasound evaluation. Panel **(C)** shows joints with PD-mode synovitis on ultrasound evaluation.

Next, the prevalence and localization of joint effusion, B- and PD-mode synovitis in MSUS were evaluated against the background of CRP-positivity, gender, type of SSc as well as prevalence of digital ulcerations. As differentiation of the degree of severity did not result in significant between-group differences (data not shown), we decided to proceed with a binary evaluation of the absence or presence of effusion and synovitis in B- and PD-mode. Briefly, CRP + individuals and males had significantly more frequently joint effusion (*p* = 0.001 and *p* < 0.00001, respectively) and B-mode synovitis (*p* < 0.00001 and *p* < 0.0001, respectively). Furthermore, patients with diffuse SSc and patients without digital ulcerations had more frequently PD-mode synovitis (*p* = 0.0052 and *p* = 0.021, respectively). See Table 3 as well as Figures 2A–C for details.

**TABLE 3:** Percentage frequency of joint effusion, B-mode synovitis, PD-mode synovitis are shown correlated with patient characteristics.

	CRP + cohort	CRP− cohort	*P*-value
**Joint effusion**	10.3% (90)	6.6% (131)	**0**.**001**
**B-mode Synovitis**	4.0% (35)	1.3% (25)	**<0**.**00001**
**PD-mode Synovitis**	1.2% (10)	0.5% (10)	0.0848
	**Female gender**	**Male gender**	***P*-value**
**Joint effusion**	6.1% (137)	13.6% (84)	**<0**.**00001**
**B-Synovitis**	1.4% (32)	4.6% (28)	**<0**.**0001**
**PD-mode Synovitis**	0.6% (13)	1.1% (7)	0.169
	**Limited SSc**	**Diffuse SSc**	***P*-value**
**Joint effusion**	6.9% (113)	8.8% (108)	0.066
**B-Synovitis**	1.5% (24)	2.9% (36)	**0**.**0081**
**PD-mode Synovitis**	0.3% (5)	1.2% (15)	**0**.**0052**
	**Digital ulcerations present**	**No digital ulcerations**	***P*-value**
**Joint effusion**	5.1% (29)	8.4% (192)	**0**.**0083**
**B-Synovitis**	1.1% (6)	2.4% (54)	0.070
**PD-mode Synovitis**	0.0% (0)	0.9% (20)	**0**.**021**

Patient characteristics: CRP-positivity, gender, type of systemic sclerosis and prevalence of digital ulcerations. Numbers are given as relative (%) and absolute numbers (numbers in brackets) of affected joints. CRP, C-reactive protein; SSc, systemic sclerosis. Bold *p*-values are significant.

Significantly more CRP + than CRP- patients showed calcifications in ultrasound examinations of their joint and ligament structures (*p* = 0.028).

In summary, arthritis was more common in SSc-patients with continuously elevated CRP levels.

### Performance of clinical examination in comparison to musculoskeletal ultrasound

In the following we outline the findings on patient level. In clinical examination 52% (34/65) of the patients showed at least one painful and/or swollen joint. In 25% (16/65) of the patients joint effusion and/or synovitis in B- and/or PD-mode could be detected in a clinically conspicuous joint. In 88% of all examined patients there was at least one effused joint detectable in MSUS (*n* = 57/65; 220 joints with effusions), 40% showed B-mode-synovitis (*n* = 26/65; 60 joints B-mode positive) and 17% were PD-positive (*n* = 11/65, 20 joints PD-positive).

In total, 2853 joints were examined both clinically and by MSUS. Fifteen percent (32/220) of joints that showed at least effusion in MSUS were conspicuous in clinical examination. 25% (5/20) of the joints that were PD-positive had arthritis in clinical examination. Overall, only 9% (32/357) of the joints that indicated pathologies (pain and/or swelling) on clinical examination showed pathological findings in MSUS. These data show that the majority of clinically conspicuous joints (91%) were not confirmed by MSUS.

In summary, in patients with arthralgia MSUS could detect clinically inapparent arthritis and was markedly superior to clinical examination in terms of sensitivity and specificity: Clinical examination showed a sensitivity of 14.6% and a specificity of 87.7%. A high mRSS > 10 significantly reduced the specificity of clinical examination to 85.2% (*p* = 0.0012). In contrast, sensitivity was only nominally reduced by the presence of soft tissue edema or a high mRSS > 10. Differentiation between limited and diffuse SSc resulted in a sensitivity of 11.3% and a specificity of 93.7% for limited SSc, and a sensitivity of 18.1% and a specificity of 79.6% for diffuse SSc.

In the case of joints that were both swollen and tender in clinical assessment the sensitivity of clinical examination decreased to 1.8% compared to MSUS.

## Discussion

First, we aimed to investigate, whether the presence of inflammatory arthritis in clinical and/or ultrasound examination is associated with elevated CRP levels in patients with SSc.

The connection between arthritis and increased CRP values in patients with SSc has already been examined by various working groups in the past (6, 7, 15, 16), however, not always using MSUS for diagnosis of arthritis but X-ray (15), which is clearly less sensitive and cannot always distinguish active from previous, currently not active arthritis.

In the present study, patients were assigned to a CRP positive or CRP negative cohort according to their consecutive CRP values over 5mg/L over the past two years allowing for a more comprehensive overview of the systemic inflammatory activity in our patients. This is in contrast to earlier studies in which CRP values were often measured only once (6, 7, 15, 16). Furthermore, in previous studies (6, 7, 15), CRP values above 10 mg/L were considered elevated, which is a cut-off value twice as high as in our study. Muangchan et al. used multiple CRP measurements, set a cut-off at 8 mg/l and found no correlation between elevated CRP values and inflammatory arthritis; these patients were however only evaluated by clinical examination alone, and not by an imaging technique (2). Lescoat et al. on the other hand, considered patients with CRP values above 5 mg/L as CRP positive, which corresponds to the reference range at the central laboratory of Freiburg University Medical Center used for our study. They described a positive correlation between pathologic joints in the MSUS examination and increased CRP values. However, the CRP values were only determined at a single point in time (16), being a mere snapshot of the inflammatory activity. Moreover, all the above mentioned studies focused on hand joints only, whereas we examined both the joints of the hands and feet in order to also map the weight-bearing joints and to provide a more comprehensive overview of potentially affected joints.

Our data show that the joints of CRP + SSc patients more frequently present inflammatory arthritis than those of CRP− SSc patients. In addition, male patients had significantly more frequently joint effusions and B-mode synovitis, and patients with diffuse SSc had significantly more often PD-mode synovitis.

The pathophysiological causes, however, need further investigation.

Earlier studies found signs of arthritis in about a quarter of patients both clinically and radiologically, which speaks against incidental occurrence of arthritis in patients with SSc (8, 17). As arthritis in SSc is comparatively frequent, and impacts significantly on joint function, as confirmed by our study, it has been discussed in the literature whether arthritis in SSc is causally related to SSc or an overlap syndrome with rheumatoid arthritis, or whether it can be seen as part of an independent disease (17–19). To reduce risk of bias and confounders, and in order to increase selectivity, we therefore excluded patients with rheumatoid arthritis, overlap syndrome, myositis or elevated rheumatoid factor and/or anti-CCP antibodies from participation in our study.

Previous studies described pathological changes in especially the MCP and PIP joints, as well as the wrists and ankles (8, 20). This is similar to our findings, which showed a particular involvement of the wrists, PIP joints, ankles and the MTP joints with B- and PD-mode positive arthritis mainly occurring in the wrists and MTP joints.

Next, we evaluated the sensitivity and specificity of clinical examination compared to musculoskeletal ultrasound (MSUS).

All patients were therefore examined clinically by joint count as well as sonographically by MSUS for the presence of arthritis. Interestingly, only a quarter of PD-positive joints showed abnormalities in clinical examination. Conversely, less than 10% of joints that were painful or swollen in clinical examination showed any kind of change in MSUS. Our findings suggest superiority of MSUS over clinical joint examination. Our results are in line with most recently published studies (6–8, 16) regarding the fact that far more pathological joints were found by MSUS, and clinical evaluation often failed to detect synovial inflammation. An advantage of our study is the larger number of joints assessed by a more sensitive imaging modality applying stringent methodology.

The above studies had in common that clinical examination missed joint involvement, but all joints identified in clinical examination showed pathologies in MSUS (6, 7). This could not be confirmed by our study. Some patients presented with signs of arthritis in clinical examination but did not have any correlates in MSUS. Other patients who were clinically unremarkable had pathological changes in MSUS. Hence, even if there is clinically no evidence of arthritis MSUS should be considered in patients reporting joint pain.

A confounding factor limiting the sensitivity of clinical examination and explaining in part the high number of painful joints without ultrasound correlates is skin thickening and puffiness/edema in SSc patients. In SSc, changes in skin can lead to severe periarticular skin tension. Patients often perceive this as joint pain. In a purely clinical examination this distinction is difficult to make. In this context, MSUS offers the possibility to differentiate whether or not there is true inflammatory arthritis.

In summary, the joints of CRP + SSc patients exhibited arthritis more often than the joints of CRP− SSc patients. The underlying pathophysiological mechanisms require further investigation. Arthritis might represent one possible cause of CRP elevation.

Given the poor sensitivity of clinical joint examination, the implementation of joint ultrasound into daily clinical routine should be considered, especially in painful joints.

There are some limitations to our study. First, standardized X-ray examinations of the joints affected were not available. Therefore, we do not have information about the erosive state or other manifestations of radiographic bone damage in this cohort, or the effects of a continuously elevated CRP might have had in these patients. Second, the subgroups of patients with tendon friction rubs (TFR) or calcinosis cutis were too small to deduct a meaningful statement from. Bearing in mind that especially TFR is usually associated with early and more severe SSc, calcinosis cutis with severe SSc, focusing on these manifestations and its connection to CRP levels might be a valid target for a consecutive study.

One of the strengths of our study is that we only enrolled patients with CRP values available over the last two years, enabling us to truly tell apart CRP + patients from CRP− patients. Furthermore, we assessed a comparatively high number of joints both clinically and ultrasonographically, which makes our study one of the largest ultrasound study in the field of SSc.

Whether CRP+ patients will benefit more than CRP- patients from immunosuppressive treatment such as methotrexate, mycophenolate or tocilizumab is an important question relevant for personalized treatment of SSc. The results of our study should be useful to design future prospective randomized trials which may address treatment stratification based on CRP levels.

## Data availability statement

The original contributions presented in this study are included in the article/Supplementary material, further inquiries can be directed to the corresponding author. Data are available upon reasonable request.

## Ethics statement

The studies involving human participants were reviewed and approved by Ethik-Kommission der Albert-Ludwigs-Universität Freiburg, Engelberger Straße 21, 79106 Freiburg E-Mail: ekfr@uniklinik-freiburg.de Telefax 0761/270 – 72630 (386/17). The patients/participants provided their written informed consent to participate in this study.

## Author contributions

DF, SF, UH, FK, and RV contributed to conception and design of the study. DF and SF organized the study, recruited patients, and performed MSUS and blood withdrawal. IJ performed the clinical examination. DF wrote the first draft of the manuscript. All authors contributed to manuscript revision, read, and approved the submitted version.

## Supplementary material

Supplementary Figure 1 Image examples of synovitis grades 0-III° in B- and PD-mode are shown for wrists, and of synovitis grades 0-II° in B- and PD-mode for MTP I joints, respectively. S1a, c, e, g and 1i, k, m, o: images show B-mode; S1b, d, f, h, and j, l, n, p: images show PD-mode. In detail for wrists: S1a, b: longitudinal plane, slight joint effusion I°, no synovitis in B-or PD-mode. S1c, d: ulnar view longitudinal, joint effusion II°, B-mode synovitis II°, PD-mode synovitis I°. S1e, f: ulnar view longitudinal; joint effusion II°; B-mode synovitis II°; PD-mode synovitis II°; peritendinitis around the extensor digitorum tendon. S1g, h: longitudinal plane; joint effusion II°; B-mode synovitis III°; PD-mode synovitis III°. R: radius; U: ulna; L: lunate; C: capitate; T: triquetrum. In detail for MTP joint I, all longitudinal plane: S1i, j: no joint effusion; no synovitis in B- or PD-mode. S1k, l: joint effusion I°; B-mode synovitis I°; no synovitis in PD-mode. S1m, n: no joint effusion; B-mode synovitis I°; PD-mode synovitis I°. S1o, p: no joint effusion; B-mode synovitis II°; PD-mode synovitis II°. MTP head: metatarsophalangeal head; PB: phalangeal base. *White asterisks indicate distinct skin thickening.
