# Supplementary figures and images for: Inflammatory arthritis in systemic sclerosis is associated with elevated C-reactive protein and requires musculoskeletal ultrasound for reliable detection
Source: Front Med (Lausanne). 2023 Jan 18;9:933809. doi: 10.3389/fmed.2022.933809 (PMC9889538; doi:10.3389/fmed.2022.933809)

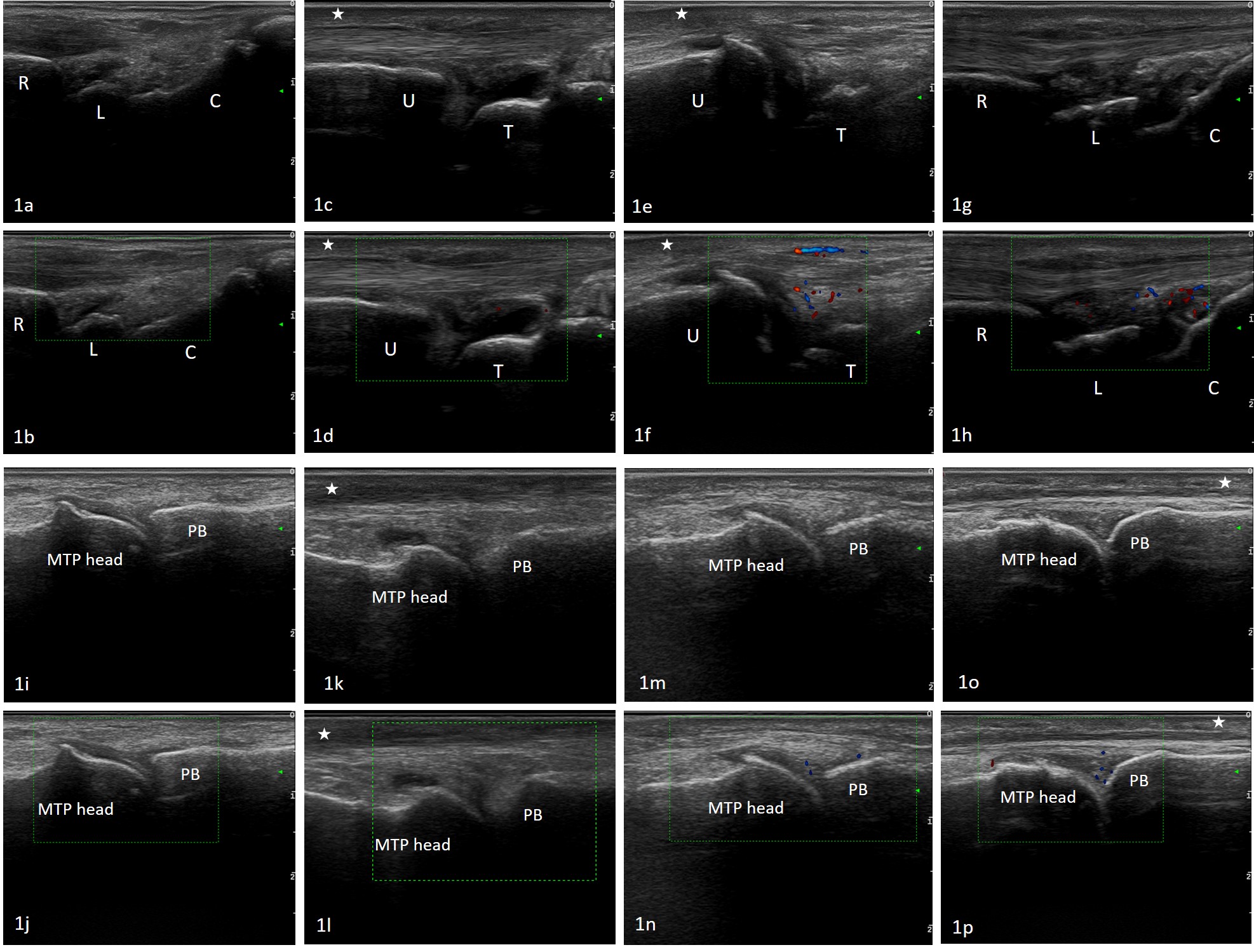

Supplement: Supplementary Figure 1 — Image examples of synovitis grades 0-III° in B- and PD-mode are shown for wrists, and of synovitis grades 0-II° in B- and PD-mode for MTP I joints, respectively. S1a, c, e, g and 1i, k, m, o: images show B-mode; S1b, d, f, h, and j, l, n, p: images show PD-mode. In detail for wrists: S1a, b: longitudinal plane, slight joint effusion I°, no synovitis in B-or PD-mode. S1c, d: ulnar view longitudinal, joint effusion II°, B-mode synovitis II°, PD-mode synovitis I°. S1e, f: ulnar view longitudinal; joint effusion II°; B-mode synovitis II°; PD-mode synovitis II°; peritendinitis around the extensor digitorum tendon. S1g, h: longitudinal plane; joint effusion II°; B-mode synovitis III°; PD-mode synovitis III°. R: radius; U: ulna; L: lunate; C: capitate; T: triquetrum. In detail for MTP joint I, all longitudinal plane: S1i, j: no joint effusion; no synovitis in B- or PD-mode. S1k, l: joint effusion I°; B-mode synovitis I°; no synovitis in PD-mode. S1m, n: no joint effusion; B-mode synovitis I°; PD-mode synovitis I°. S1o, p: no joint effusion; B-mode synovitis II°; PD-mode synovitis II°. MTP head: metatarsophalangeal head; PB: phalangeal base. *White asterisks indicate distinct skin thickening. [file Image_1.jpeg]
